# Vaccine-induced neutralizing antibodies against SARS-CoV-2 Omicron variant isolated in Osaka, Japan

**DOI:** 10.1099/acmi.0.000465.v3

**Published:** 2023-02-27

**Authors:** Satoshi Hiroi, Saeko Morikawa, Kazushi Motomura, Haruyo Mori

**Affiliations:** ^1^​ Division of Microbiology, Osaka Institute of Public Health, Osaka, Japan; ^2^​ Division of Public Health, Osaka Institute of Public Health, Osaka, Japan

**Keywords:** neutralizing antibody, SARS-CoV-2, vaccine

## Abstract

To study vaccine-induced neutralizing antibodies against severe acute respiratory syndrome coronavirus 2 (SARS-CoV-2) variants isolated in Osaka, Japan, microneutralization tests were performed on serum samples from 32subjects who received a second dose of vaccination, and 10 of those who received the third dose of vaccination. Geometric mean titres (GMTs) for the D614G strain, Alpha variant, Delta variant, and Omicron BA.1 of the subjects after the second dose of vaccination were 19.5, 21.8, 6.3 and 2.0, respectively. The GMT for the Delta variant was significantly lower than that for the D614G strain and Alpha variant, and the GMT for the Omicron BA.1 was significantly lower than that for the Delta variant. Among the subjects who received three doses of vaccination, the GMTs for the Omicron BA.1 (62.8) and BA.2 (38.6) were significantly higher than that for the Omicron BA.1 after the second dose. Thus, in the present study, the second dose of vaccination induced neutralizing antibodies against SARS-CoV-2 strains, and the reactivity of neutralizing antibodies to the variants was thought to be enhanced by the third dose of vaccination. The serum samples used in this study will be useful in evaluating the reactivity of vaccine-induced antibodies to newly emerging variants.

## Data summary

All data on neutralization titres in this study are available in the figshare repository (https://doi.org/10.6084/m9.figshare.21959621 [1]).

## Introduction

Severe acute respiratory syndrome coronavirus 2 (SARS-CoV-2), the novel coronavirus that causes COVID-19, was first identified at the end of 2019 and has since spread to spawn a worldwide pandemic. SARS-CoV-2 variants have emerged in numerous countries including Japan, which has experienced five waves of the COVID-19 pandemic as of November 2021 [2]. The second and third waves, which began in July and November 2020, respectively, were mainly caused by variants carrying the D614G mutation in the spike protein (B.1.1.214 and B.1.1.284), which enhances infectivity [3]. In the fourth wave, which began in March 2021, the Alpha variant (B.1.1.7) was dominant, especially in Osaka [4]. Meanwhile, the fifth wave, which began in July 2021, was predominated by the highly transmissive Delta variant (B.1.617.2) [5], and the sixth wave, which began in January 2022, was predominated by the highly mutated Omicron variant (B.1.1.519) BA.1 and BA.2 [6].

Vaccines play an important role in the acquisition of immunity to SARS-CoV-2, with several studies having reported their effectiveness against COVID-19 [7, 8]. In Japan, vaccinations against COVID-19 started in February 2021, and a third dose is also being administered in 2022 [9]. Serum neutralizing antibody titres can be used to predict vaccine efficacy against SARS-CoV-2 variants of concern [10]. Given that studies on the third dose of vaccine-induced neutralizing antibodies against SARS-CoV-2 variants remain limited in Japan, we used serum samples from vaccinated subjects and measured antibody titres against isolated strains in Osaka using the microneutralization method.

## Methods

Blood samples were collected at the Osaka Institute of Public Health for the national epidemiological surveillance of vaccine-preventable diseases. Residual serum from those who had received a second dose of a BNT162b2 mRNA vaccine (Pfizer–BioNTech), which encodes the SARS-CoV-2 (Wuhan-Hu-1 strain) full-length spike protein [11], at least three weeks prior were used as samples. In addition, blood samples were collected at least 3 weeks after the third dose of the COVID-19 mRNA vaccine from subjects who provided written informed consent.

The serum samples were inactivated for 30min at 56°C to eliminate non-specific inhibitors. Five virus strains, the D614G strain (B.1.1.284; hCoV-19/Japan/OIPH16/2020), Alpha variant (B.1.1.7; hCoV-19/Japan/OIPH18/2021), Delta variant (B.1.617.2; hCoV-19/Japan/OIPH30/2021) and Omicron variant (BA.1; hCoV-19/Japan/OIPH39/2021, BA.2; hCoV-19/Japan/OIPH2/2022) were isolated in our biosafety level 3 laboratory using VeroE6/TMPRSS2 cells (JCRB 1819) [12].

For microneutralization tests, serially diluted serum samples starting from a 1 : 5 dilution were mixed with an equal volume of viral solution containing the 50% tissue culture infective dose (100 TCID_50_/50 µl) (final dilution range 1 : 10 to 1 : 1280). The serum-virus mixture was incubated for 1h at 37°C, after which 50µ l of the mixture at each dilution was added to VeroE6/TMPRSS2 cells with 50µl of Dulbecco’s Modified Eagle Medium (Sigma, St. Louis, MO, USA) containing 2% fetal bovine serum in 96-well plates. The plates were incubated for 4days at 37°C. Subsequently, the neutralization titre was determined using the endpoint method from the highest dilution at which more than 50 % of cells were protected based on observed cytopathic effects. The microneutralization tests were conducted in duplicate, and for calculation of geometric mean titre (GMT), titre values below 10 were assigned as a value of 1 [13]. Statistical analyses of antibody titres between groups (by variants) were performed using Kruskal–Wallis and Mann–Whitney tests, and Bonferroni adjustment was applied. The correlation between age and antibody titres was evaluated using Spearman’s correlation coefficient. Differences in age and gender ratio between two-dose and three-dose vaccination groups were determined by Mann–Whitney test and Fisher’s exact test, respectively. SPSS 16.0 software (SPSS, Chicago, IL, USA) was used for the analysis.

## Results

Serum samples from 32subjects who received two doses of vaccine (age 28 to 67years, mean age 44.0years, percent male 56.3 %) and 10 of those who received three doses of vaccine (age 30 to 64years, mean age 46.7years, percent male 60.0 %) were examined for neutralization of SARS-CoV-2 variants in this study (Table S1, available in the online version of this article). Comparison of the two-dose and three-dose vaccination groups showed no significant differences in age (*P*=0.44) and gender ratio (*P*=1). All subjects received the BNT162b2 mRNA COVID-19 vaccine, and blood samples were collected 3–12weeks after the second dose and 3–11 weeks after the third dose. Mutations in the SARS-CoV-2 spike protein of the D614G strain, Alpha, Delta and Omicron variants compared to the vaccine strain are outlined in Table 1 [14, 15].

**Table 1.** Mutations of spike protein of the SARS-CoV-2 variants compared to the Wuhan-Hu-1 strain. Bold letters indicate mutations in receptor-binding domain

Variant	Mutations
D614G strain	D614G
Alpha	Δ69–70, Δ144, **N501Y**, A570D, D614G, P681H, T716I, S982A, D1118H
Delta	T19R, Δ156–157, R158G, **L452R, T478K**, D614G, P681R, D950N
Omicron BA.1	A67V, Δ69–70, T95I, G142D, Δ143–145, Δ211, L212I, 214EPE, **G339D**, **S371L**, **S373P**, **S375F**, **K417N**, **N440K, G446S**, **S477N**, **T478K**, **E484A**, **Q493R**, **G496S**, **Q498R**, **N501Y**, **Y505H**, T547K,D614G, H665Y, N679K, P681H, N764K, D796Y, N856K,Q954H, N969K, L981F
Omicron BA.2	T19I, Δ24–26, A27S, G142D, V213G, **G339D**, **S371F**, **S373P**, **S375F**, **T376A**, **D405N**, **R408S**, **K417N**, **N440K**, **S477N**, **T478K**, **E484A**, **Q493R**, **Q498R**, **N501Y**, **Y505H**, D614G, H665Y, N679K, P681H, N764K, D796Y, Q954H, N969K

**Fig. 1.**
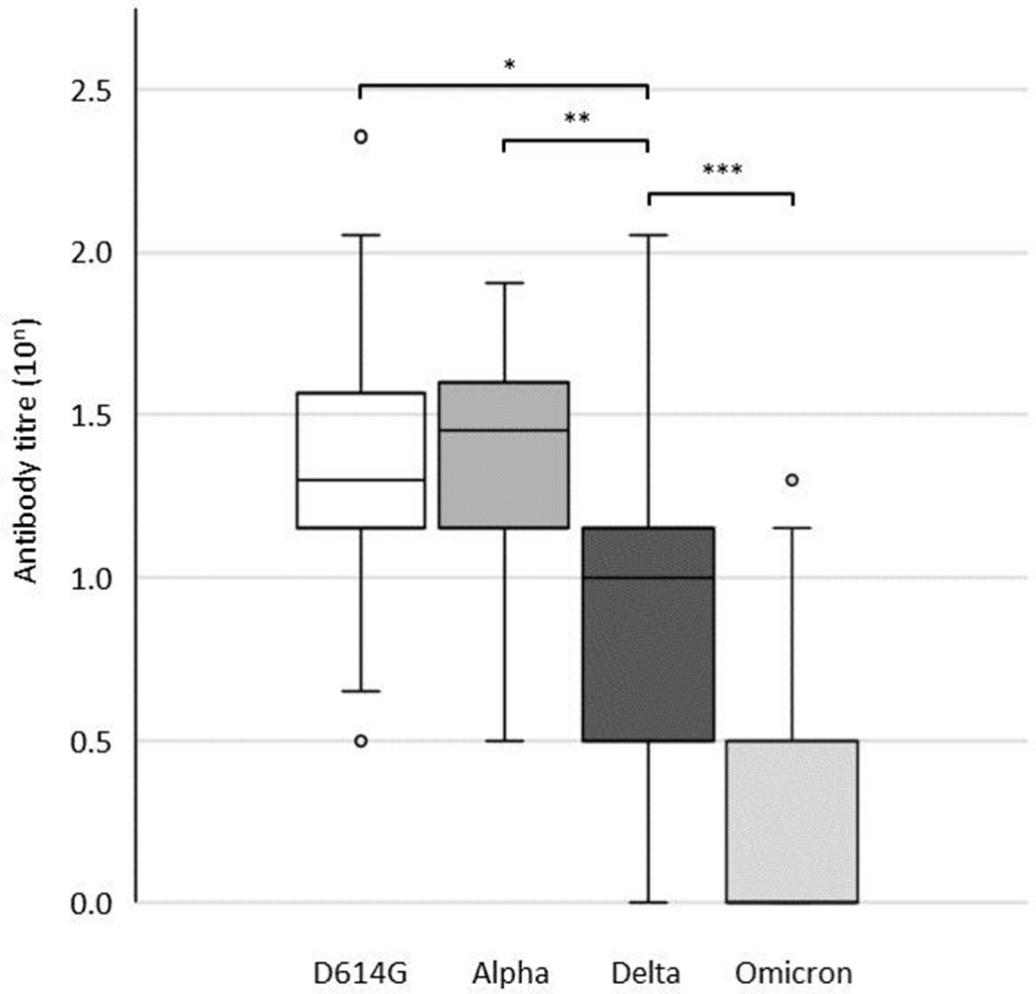
Comparison of the geometric mean antibody titres against SARS-CoV-2 D614G strain, Alpha, Delta, and Omicron BA.1 variants in 32 double vaccinated subjects. Box plots display the median and interquartile range. The lower and upper whiskers represent the smallest and largest values within 1.5 interquartile ranges from the first and third quartile. Mann–Whitney analyses with Bonferroni’s correction were used to investigate differences between pairs of variants. (*P <0.0005, **P <0.00005, ***P <0.005).

After two doses of vaccination, the GMTs for the D614G strain, Alpha variant, Delta variant and Omicron BA.1 in the subjects were 19.5, 21.8, 6.3 and 2.0, respectively (Fig. 1). There was no statistically significant difference (*P*=0.36) between the GMTs for the D614G strain and the Alpha variant. In contrast, the GMT for the Delta variant was statistically significantly lower than that for the D614G strain (*P* <0.0005) and Alpha variant (*P* <0.00005), and the GMT for the Omicron BA.1 was significantly lower than that for the Delta variant (*P* <0.005). There was no correlation between age and antibody titre against any of the virus strains with a Spearman correlation coefficient range from −0.077 to −0.036. Likewise, there was no difference in the antibody titre to each strain based on gender (*P*-value range from 0.42 to 0.89).

**Fig. 2.**
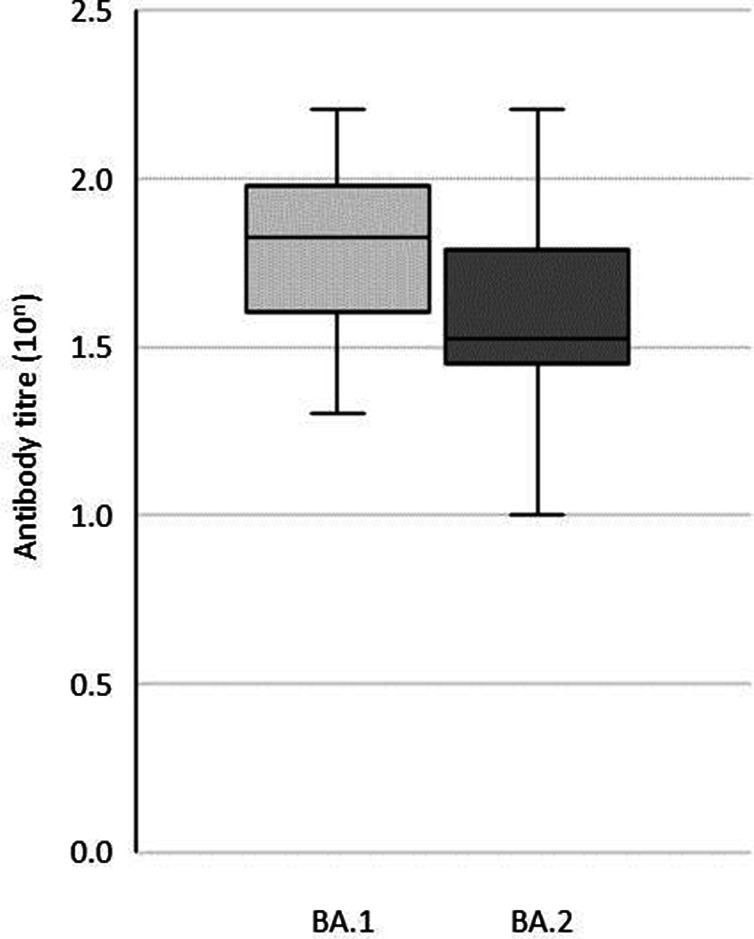
Comparison of the geometric mean antibody titres against Omicron BA.1 and BA.2 in 10 triple vaccinated subjects. Box plots display the median and interquartile range. The lower and upper whiskers represent the smallest and largest values within 1.5 interquartile ranges from the first and third quartile.

Fig. 2 shows the antibody titres of the subjects who received the third dose of vaccination. The GMTs for the Omicron BA.1 and BA.2 were 62.8 and 38.6, respectively, and the GMT to BA.2 was lower than that to BA.1, but the difference was not statistically significant (*P*=0.11). The GMTs against Omicron BA.1 and BA.2 were significantly increased (*P*<0.000005) after the third dose of vaccination compared to the GMT for the Omicron BA.1 after the second dose.

## Discussion

In the present study, we evaluated vaccine-induced antibodies to circulating SARS-CoV-2 variants using a serum neutralization test. Compared to the D614G strain and Alpha variant, the subjects after the second dose of vaccination had lower neutralizing activity against the Delta variant and Omicron BA.1, which is consistent with reports that immune evasion by the Delta and Omicron variant reduces the sensitivity of vaccine-elicited antibodies [15–17]. Unlike the second dose, the third dose of vaccination induced antibodies with neutralizing activity against the Omicron BA.1 and BA.2. These results support previous study regarding the efficacy of booster dose vaccination against the Omicron variant [18]. Although vaccines may not be completely effective for preventing SARS-CoV-2 infection, they are effective against the variants for preventing hospitalization and death [19, 20]. It implies that vaccines can stimulate not only humoral immunity but also cellular immunity [21, 22].

Since none of the 32subjects who received two doses of vaccine had a history of COVID-19 infection, we assumed that all of the subjects acquired antibodies from vaccination. While previous studies have reported age- and gender-related differences in immune response [23], we could not find the correlation between antibody titre and age or gender. This discrepancy may be due to the limitations of this study, namely the small number of subjects and the inclusion of multiple periods between vaccination and blood collection. Future studies on the seroprevalence of SARS-CoV-2 need to have larger sample sizes and investigate cellular immune responses as well as humoral immune responses.

Our results showed that GMTs against Omicron BA.1 and BA.2 after the third dose increased substantially compared to that after the second dose. The increased reactivity of neutralizing antibodies produced by repeated vaccination against the Omicron variant is thought to be affinity maturation and recall of broadly neutralizing antibodies, as have been reported [24, 25]. In this study, antibody titres to Omicron BA.2 were slightly lower than those to BA.1. Differences in neutralizing antibody titres to BA.1 and BA.2 varied among reports, but their differences in antigenicity are considered to be limited [14, 17].

## Conclusions

The present study demonstrated that the vaccination induced neutralizing antibodies against SARS-CoV-2 strains, and the third dose of vaccination enhanced the reactivity of the neutralizing antibodies to the Omicron variants. Because the effectiveness of the vaccine has been reported to wane over time [26], continuous investigation of antibody levels will be needed. Despite Japan’s sixth wave of infection having declined in June 2022, it is important to continue to monitor the recurrence of the Omicron variants including BA.4 and BA.5, and the emergence of new yet-to-be-detected variants. The serum samples used in this study may be useful for evaluating the reactivity of vaccine-induced antibodies to newly emerging variants.

## Peer review history

### VERSION 2

#### Editor recommendation and comments

https://doi.org/10.1099/acmi.0.000465.v2.1

© 2022 Rudkin J. This is an open access peer review report distributed under the terms of the Creative Commons Attribution License.

**Justine Rudkin**; University of Oxford, Nuffield Department of Population Health, UNITED KINGDOM, Oxford

Date report received: 28 November 2022

Recommendation: Accept

**Comments**: Thank you for addressing the reviewers comments and adding in the table detailing the spike protein mutations of each variant. This manuscript is now suitable for publication, with one caveat- all raw data including technical replicates need to be provided as part of our open data policy. The table you have provided as a supplementary S1. only gives a single average data point for each sample. The materials and methods state each sample was assessed in duplicate. Therefore, please deposit all raw data underlying the work in the Society’s data repository Figshare account here: https://microbiology.figshare.com/submit. Please also cite this data in the materials and methods of the main manuscript and list it as a unique reference in the References section. When you resubmit your article, the Editorial staff will post this data publicly on Figshare and add the DOI to the Data Summary section where you have cited it. This data will be viewable on the Figshare website with a link to the manuscript and vice versa, allowing for greater discovery of your work, and the unique DOI of the data means it can be cited independently should it be used by other researchers.

#### Author response to reviewers to Version 1

Response to Reviewer 1

We wish to thank you for your valuable comments. We agree with you and have revised our paper in accordance with your comments.

1. The sample size of this paper is very limited and the study only evaluated the neutralization antibody response. There was no attempt to measure the overall antibody response or to examine cell mediated immunity. Realize that this was not the purpose of the study but a large sample size, more frequent sampling of the subjects and more expanded analysis of the immune response would have been a stronger paper.

Response to comments

As you have pointed out, we understand that the sample size of our study is not sufficient. We are considering to increase the number of subjects from whom samples are collected when analyzing immune responses to SARS-CoV-2 in the future. Therefore, we have added the sentence “Future studies on the seroprevalence of SARS-CoV-2 need to have larger sample sizes and investigate cellular immune responses as well as humoral immune responses.” to the limitations of this study. Please see line 144-145 of the revised manuscript.

Again, we appreciate all of your insightful comments. Thank you for taking the time to help us improve the paper.

Response to Reviewer 2

We wish to thank you for your valuable comments. We have incorporated your suggestion and revised our paper.

1. Line 68, [encoding the SARS-CoV-2 full-length spike] is suggested to be added after [BNT162b2 mRNA vaccine].

Response to comments

As you suggested, we have revised sentences as “Residual serum from those who had received a second dose of a BNT162b2 mRNA vaccine (Pfizer–BioNTech), which encodes the SARS-CoV-2 (Wuhan-Hu-1 strain) full-length spike protein, at least three weeks prior were used as samples.” in line 66-69.

2. Please add a figure or table to outline the differences of various SARS-CoV-2 variants [including the vaccine strain] in the spike gene. Alternatively, cite a reference to mention the differences of these variants in the spike gene.

Response to comments

We have added a table to show the spike protein mutations of the D614G strain, Alpha, Delta and Omicron (BA.1, BA.2) variants as Table 1, and described about the table in line 105-107.

3. Lines 101, 102, 106, please write down the p values to show they are not statistically important.

Response to comments

We have added *P*values and revised the sentences. Please see line 110-111 and 114-117 of the revised manuscript.

4. In this study, antibody titers of two different groups [with two doses, with three doses] were compared. Please compare these two groups, is there any difference in main demographic characteristics, such as gender, age, etc.

Response to comments

Additional statistical analyses between the two groups were carried out and the results were described in revised manuscript (line 102-103). The methods of the statistical analyses have been revised and are described in line 89-95 of the materials and methods section.

Again, we appreciate all of your insightful comments. Thank you for taking the time to help us improve the paper.

### VERSION 1

#### Editor recommendation and comments

https://doi.org/10.1099/acmi.0.000465.v1.5

© 2022 Rudkin J. This is an open access peer review report distributed under the terms of the Creative Commons Attribution License.

**Justine Rudkin**; University of Oxford, Nuffield Department of Population Health, UNITED KINGDOM, Oxford

Date report received: 27 September 2022

Recommendation: Minor Amendment

**Comments**: I would like to thank both reviewers for their giving their time and expertise to assess this manuscript- it is appreciated. Thank you also to the authors for patiently waiting for this manuscript to be moved through the peer review system, which I acknowledge has taken considerable time. Both reviewers have commented that the methodology is sound and that the conclusions are supported by the data. Whilst I acknowledge the comments of reviewer 1 about the size and scope of the study and the limitations this places on the data, Access Microbiology operates a sound science policy with methodological rigour outweighing impact and novelty. Therefore, my decision is for minor amendments to be made to improve the manuscript before it can be accepted for publication. Can the authors please make the amendments suggested by reviewer 2. The addition of a figure or table outlining the differences in the spike gene between variants would certainly improve the manuscript. In line with Access Microbiology’s Open Data policy authors also need to deposit all of the raw data analysed in this study into an open access repository, or include it as a supplementary table in the manuscript so that it can be accessed by other researchers. https://www.microbiologyresearch.org/open-data

#### Reviewer 2 recommendation and comments

https://doi.org/10.1099/acmi.0.000465.v1.3

© 2022 Lo S. This is an open access peer review report distributed under the terms of the Creative Commons Attribution License.

**Shih-Yen Lo**; Tzu Chi University, Laboratory Medicine and Biotechnology, 701, Sec. 3, Chung Yang Road, Hualien, TAIWAN, -886-3-8571917

https://orcid.org/0000-0001-8651-0148

Date report received: 27 September 2022

Recommendation: Minor Amendment

**Comments**: This study intends to investigate the vaccine-induced neutralizing antibodies against severe acute respiratory syndrome coronavirus 2 (SARS-CoV-2) variants isolated in Osaka, Japan. Microneutralization tests were performed on serum samples from 32 subjects who received two doses of vaccination and 10 subjects with three doses. Among those with two doses, the Geometric Mean Titres (GMT) for the Delta variant was significantly lower than that for the D614G strain and Alpha variant, and the GMT for the Omicron BA.1 was significantly lower than that for the Delta variant. Among those with three doses, the GMTs for the Omicron BA.1 and BA.2 were significantly higher than that for the Omicron BA.1 in those with two doses. Several suggestions: 1. Line 68, [encoding the SARS-CoV-2 full-length spike] is suggested to be added after [BNT162b2 mRNA vaccine]. 2. Please add a figure or table to outline the differences of various SARS-CoV-2 variants [including the vaccine strain] in the spike gene. Alternatively, cite a reference to mention the differences of these variants in the spike gene. 3. Lines 101, 102, 106, please write down the p values to show they are not statistically important. 4. In this study, antibody titers of two different groups [with two doses, with three doses] were compared. Please compare these two groups, is there any difference in main demographic characteristics, such as gender, age, etc.

*Please rate the manuscript for methodological rigour*

Very good

*Please rate the quality of the presentation and structure of the manuscript*

Satisfactory

*To what extent are the conclusions supported by the data?*

Partially support

*Do you have any concerns of possible image manipulation, plagiarism or any other unethical practices?*

No

*Is there a potential financial or other conflict of interest between yourself and the author(s)?*

No

*If this manuscript involves human and/or animal work, have the subjects been treated in an ethical manner and the authors complied with the appropriate guidelines?*

Yes

#### Reviewer 1 recommendation and comments

https://doi.org/10.1099/acmi.0.000465.v1.4

© 2022 Benfield D. This is an open access peer review report distributed under the terms of the Creative Commons Attribution License.

**David Benfield**; The Ohio State University OARDC: The Ohio State University Ohio Agricultural Research and Development Center, Animal Sciences - Food Animal Health Program, 1680 Madison Avenue, Wooster, UNITED STATES

https://orcid.org/0000-0001-6492-3830

Date report received: 19 July 2022

Recommendation: Major Revision

**Comments**: This paper is a limited study of 32 subjects vaccinated twice with the commercial COVID vaccines and 10/32 that received a second booster. The goal was to determine the neutralizing antibody response to four variants of SARS CoV2. The results indicate that the vaccinates did respond to all four variants with the initial vaccination and subsequent booster, but the level of response varied depending on the isolate of SARS CoV 2 used as the target in the neutralization assay. The results are presented well. The methodology, results and conclusions agree. The sample size of this paper is very limited and the study only evaluated the neutralization antibody response. There was no attempt to measure the overall antibody response or to examine cell mediated immunity. Realize that this was not the purpose of the study but a large sample size, more frequent sampling of the subjects and more expanded analysis of the immune response would have been a stronger paper.

*Please rate the manuscript for methodological rigour*

Good

*Please rate the quality of the presentation and structure of the manuscript*

Good

*To what extent are the conclusions supported by the data?*

Strongly support

*Do you have any concerns of possible image manipulation, plagiarism or any other unethical practices?*

No

*Is there a potential financial or other conflict of interest between yourself and the author(s)?*

No

*If this manuscript involves human and/or animal work, have the subjects been treated in an ethical manner and the authors complied with the appropriate guidelines?*

Yes

#### SciScore report

https://doi.org/10.1099/acmi.0.000465.v1.1

© 2022 The Authors. This is an open-access article report distributed under the terms of the Creative Commons License.

#### iThenticate report

https://doi.org/10.1099/acmi.0.000465.v1.2

© 2022 The Authors. This is an open-access article report distributed under the terms of the Creative Commons License.

## Supplementary Data

Supplementary material 1
